# What Are the Core Competencies of a Mental Health Nurse? Protocol for a Concept Mapping Study

**DOI:** 10.3390/nursrep10020018

**Published:** 2020-12-07

**Authors:** Nompilo Moyo, Martin Jones, Rachel Cardwell, Richard Gray

**Affiliations:** 1 School of Nursing and Midwifery, La Trobe University, Bundoora, VIC 3086, Australia; R.Cardwell@latrobe.edu.au (R.C.); R.Gray@latrobe.edu.au (R.G.); 2 Victorian Tuberculosis Program, Melbourne, VIC 3000, Australia; 3 Department of Rural Health, University of South Australia, North Terrace, Adelaide, SA 5000, Australia; Martin.Jones@unisa.edu.au

**Keywords:** mental health nurse, competencies, concept mapping, users, psychiatrist

## Abstract

This study aims to identify and contrast key stakeholder perspectives about the core competencies of mental health nurses. Mental health nurses provide much of the direct care and treatment to patients with mental disorders. The perspectives of users of mental health services, mental health nurses, mental health nurse clinical leaders, psychiatrists, and mental health nurse academics regarding the core competencies of a mental health nurse are informative to improve the quality of care given to patients. We will use concept mapping to compare and contrast the views of different stakeholder groups (*n* = 50, 10 per group) about the core competencies (knowledge, skills, and attitudes) of mental health nurses. There are six stages in concept mapping: preparation, generation of statements, structuring of statements, representation of statements, interpretation of maps, and utilisation of maps. The Good Reporting of A Mixed Methods Study (GRAMMS) checklist will guide this study. The final output is a “concept map” that can be used and interpreted to understand core mental health nursing competencies. This study will provide insight into the perceived core competencies of mental health nurses from a variety of perspectives.

## 1. Introduction

Clinical competencies can be defined as a combination of knowledge, clinical skills, and attitudes [1]. Mental health nurses are registered nurses that have had specific psychiatric training either at an undergraduate or postgraduate level. There is a lack of clarity about what the core competencies of a mental health nurse are. For example, some Australian authors have argued that mental health nurses need to be expert in trauma-informed care [2], despite a paucity of evidence that this is an effective way of working [3]. In England, there has been a strong emphasis on enhancing the physical health of people with severe mental illness as a core focus of the work of mental health nurses [4,5].

Mental health nurses provide care and treatment to patients in a variety of inpatient and community settings [6,7]. The majority of the mental health nurse’s working day should be spent providing direct patient care [8]. The competencies which nurses can apply during their time with patients may impact clinical outcomes [1,9].

### 1.1. Patient Perceptions of Mental Health Nurses’ Core Competencies

Bee et al. [10] conducted a systematic review that examined service user views and expectations of registered mental health nurses. The authors included 92 qualitative and 40 observational studies that reported service user views about mental health nurse competencies. Included studies generally had a high risk of bias. Bee et al. [10] reported that the patients expect mental health nurses to fulfil a multi-faceted role in which practical and social support are provided alongside the delivery of more formal psychological interventions. Patients perceived nurses to be professional experts with the necessary skills and training to diagnose, prioritise mental health problems, and respond effectively to deteriorations in their mental health state. The majority of inpatients felt that mental health nurses were inaccessible and spent insufficient time providing direct care. Patients also perceived that nurses lacked collaborative skills; for example, patients were not involved in the planning of their treatment. The positive personal qualities of nurses reported by patients included empathy, respect, and compassion. Being dismissive or judgmental and adopting a coercive approach were identified as undesirable nursing attributes by patients.

### 1.2. Perceptions of Mental Health Nurses about Their Core Competencies

Delaney and Johnson [11] reported a meta-synthesis of the qualitative literature about mental health nurses’ perceptions of the core aspects of their role. Sixteen studies were included in the synthesis. Three themes were reported: 1. the nurses’ efforts to forge engagement with patients, 2. activities which maintained the safety of the unit, and 3. interventions which nurses viewed as educating/empowering patients. The reported observations are limited by the qualitative nature of the study design, which means that observations cannot be generalised beyond the included participants.

### 1.3. Gap in Knowledge

Previous research has focused on patient and nurse perceptions of the core competencies of mental health nurses. There are other stakeholder groups whose work is interdependent with that of clinical mental health nurses, who will have important views on their core competencies. Psychiatrists, for example, work closely with mental health nurses around the prescribing and administration of psychotropic medication. Nurse managers tend to be focused on patient safety issues; as a consequence, they may have different views on nurse competencies. Mental health nurse academics are another group that will have a further perspective that should be informed by a more contemporary understanding of the evidence base. Understanding mental health nursing competencies from these multiple stakeholders, we argue, may inform debate about curriculum development for this group of workers.

## 2. Materials and Methods

### 2.1. Concept Mapping

We will use a genuine mixed-method design. Concept mapping will be used to identify and contrast the perceptions of five stakeholder groups—users of mental health services, mental health nurses, mental health nurse clinical managers, psychiatrists, and mental health nurse academics—about their perceptions of the core competencies of mental health nurses. Concept mapping is a type of structured conceptualisation method used to organise and represent ideas from identified populations [12]. We will combine qualitative interviews with multivariate statistical analyses to describe views of the stakeholders on the core competencies of mental health nurses and visually represent these ideas as “concept maps” [13,14].

The reporting of this study will adhere to the guidelines for Good Reporting of A Mixed Methods Study (GRAMMS) [15] (refer to Supplementary File S1).

### 2.2. Why Concept Mapping?

Concept mapping has been extensively used in a variety of settings, including community mental health and public health [16,17]. It is a useful methodological approach to understanding complex phenomena. In health research, it is emerging as a novel methodological approach. It has been used to develop a deeper understanding of medication adherence [18] and why people do not access psychological services [19]. One method cannot adequately identify and compare the views of multiple stakeholders about the competencies of mental health nurses. Understanding the core competencies of mental health nurses is a complex topic that lends itself well to concept mapping as a methodology.

## 3. Study Setting and Population

This study will be conducted in the state of Victoria, Australia. We will recruit and collect data from five participant groups: users of mental health services, mental health nurses, nurse managers, psychiatrists, and nurse academics.

All participants will be aged 18 years or over, able to communicate comfortably in English, and have access to and can use a personal computer. Additionally, we will apply the following inclusion criteria for each stakeholder group:

- Users of mental health services;
- Have personal experience of mental health problems;
- Mental health nurses;
- Licensed to practice as a registered nurse in Australia and currently working in a mental health setting;
- Clinical managers;
- Licensed to practice as a registered nurse in Australia and working in a mental health setting in a leadership role (manager, team leader or director of nursing);
- Psychiatrists;
- Licensed to practice as a psychiatrist in Australia and currently working in a mental health setting;
- Mental health nurse academics;
- Working in a School of Nursing (or equivalent) in an Australian University and self-defines as a specialist in mental health.

## 4. Sample Size

We intend to recruit approximately 50 participants (ten in each participant group). There is no agreed limit on the number of participants required for concept mapping. Rosas and Kane [12] recommend that a minimum of 20 participants should complete the clustering task. Ariadne [20] reported that the minimum number of participants for concept mapping is 8–10, but there is no limit to the maximum number of participants.

## 5. Recruitment

### 5.1. Users of Mental Health Services

Convenience and snowballing techniques will be used to recruit users of mental health services through consumer advocacy groups. Potential participants will initially be contacted by telephone and/or email by a member of the researcher team. Research objectives, how the participant will be involved in the study, and the potential benefits and risks of participating in the study will be explained. Written information and a consent form will be sent (by email) to potential participants. If they agree to take part, participants will be asked to email a scanned or photographed copy of the signed consent form to the research team. Participants will be encouraged to contact the research team by phone or email if they have any additional questions about the study.

### 5.2. Professional Groups

Mental health nurses, mental health nurse clinical leaders, psychiatrists, and mental health nurse academics will be recruited via social media (Twitter, Facebook, LinkedIn). Additionally, we will use snowballing techniques, asking participants for contacts that might be interested in participating in building our sample. Potential participants can contact the research team by email or telephone to express interest in the study. They will be contacted by a study researcher, who will explain the study and send written information about the research. As with users, professional participants will be asked to send a photographed copy of the consent form via email or text message.

## 6. Scheduling Times for Concept Mapping Task

A study researcher will schedule convenient dates and time with participants for each of the concept mapping activities: brainstorming and prioritising and clustering. All research activities will be completed online using video conferencing technology.

## 7. Demographic Data

The following demographic information will be collected for all participants: 1. age in years, 2. gender (male/female/other), 3. highest academic qualification (high school/undergraduate/graduate/postgraduate).

From users of mental health services, we will additionally record 1. type of mental disorder (common (e.g., depression, anxiety) or severe (e.g., schizophrenia, bipolar)), 2. approximate duration of mental health problem (in years), 3. ever hospitalised (yes/no), 4. employment status (employed/unemployed).

We will ask the professional groups to provide the following information: 1. The country where the primary clinical qualification was awarded, 2. Years of experience working in clinical practice.

## 8. Ethics Considerations

Ethical approval will be sought from the participating university’s human research ethics committee before starting the study (Approval No. [HEC20257]). There are ethical issues with this study that require consideration. First, consent will be obtained electronically. Second, interviews will be conducted using video conferencing technology. Links to video interview will be password protected (to prevent so-called “video bombing”). We will audio record interviews that will be retained for data analysis. The recorded interviews will be deleted after data analysis.

It is possible, albeit unlikely, that participants may be known to the researcher because they have worked with the researcher in previous roles. Consequently, they may perceive that there is an expectation that they will participate in the research. We will acknowledge this issue with potential participants and emphasise the voluntary nature of taking part.

### 8.1. Risks Associated with Taking Part in the Study

Participation in any research study is associated with risk, however minimal [21]. We anticipate that some participants may have had previous negative experiences associated with psychiatric hospital admission, and being asked to talk about the attitudes, skills, and knowledge of mental health nurses may cause distress. If this occurs, we will stop the interview and check if the participant would like to take “time out,” postpone the interview, or consider withdrawing from the research. A member of the research team will be available to provide psychological support to the participant if necessary. Additionally, we will advise participants about online psychosocial support and counselling services that are available (for example, au.reachout.com, beyond blue—https://www.beyondblue.org.au).

### 8.2. Risk Mitigation during the COVID-19 Pandemic

The fieldwork for this study will be conducted during the COVID-19 pandemic. COVID-19 is a highly infectious disease caused by SARS-COV2 [22]. In Australia, during the pandemic, people are required to physically distance and engage in other infection control measures to reduce transmission [23]. Consequently, interviews will be conducted using video conferencing.

### 8.3. Potential Benefits of This Research

We do not anticipate any direct benefits to participants from being involved in the study. We anticipate that the findings from this research will inform the curriculum of mental health nurse training internationally, with the ultimate aim of ensuring that psychiatric nurses have the required skills to work with people experiencing mental illness.

## 9. Data Collection Procedure

Concept mapping requires participants to take part in a “brainstorming” interview and complete a series of online computer-based tasks (prioritising and clustering). There are six discreet phases in the concept mapping process [13]:

### 9.1. Phase 1: Preparation

In the preparation phase, the task is to decide on the focus question and identify participant groups. The focus of this study is, “what are the core competencies (knowledge, clinical skills, and attitudes) of a mental health nurse?” We will seek to elicit the views of users of mental health services, psychiatric nurses, clinical leaders, psychiatrists, and nurse academics.

### 9.2. Phase 2: Generation of Statements (Brainstorming) (via Video Conferencing)

Interviews will be conducted using video conferencing technology. We will audio record interviews, which will be retained for data analysis. Participants will be interviewed individually and asked to generate statements in response to the following question: “What are the most important competencies (knowledge, skills, and attitudes) of a mental health nurse?” Participants will be asked to give simple statements and avoid jargon. Participants will be asked to try and avoid long sentences, use conditional formulations (if, then), make comparisons (e.g., “is better than”), use double negatives, make value judgements (“I think it would be good if”) [20]. Listening back to the interview, a researcher will extract individual statements that are singular, specific, and abstract (interviews are not transcribed in concept mapping). If we generate more than 98 statements, we will need to combine repetitive or overlapping concepts into single statements, as this is the maximum number that can be entered into the ARIADNE software package [20].

### 9.3. Phase 3: Structuring of Statements (Completed Online)

Structuring of statements will consist of two tasks: prioritising and clustering. Both tasks will be completed online using the ARIADNE software package. Participants will be emailed a link to ARIADNE software to enable them to participate.

#### 9.3.1. Phase 3a: Prioritisation

Participants will be asked to rank statements in order of importance (i.e., what are the most essential competencies of the mental health nurse), from 1 (least important) to 5 (most important). Each point on the scale must have an equal number of statements. Participants will be advised not to consider the lowest category “unimportant” but “least important” to avoid a situation where participants refuse to place statements in the lowest category because they still consider them important [20].

#### 9.3.2. Phase 3b: Clustering

Participants will be asked to group statements that seem to belong together into clusters. Clusters are not required to have equal numbers of statements. Participants can give clusters names if they chose to do so (this is not a required part of the process). Participants will follow the following rules: each statement must be included in a cluster; each statement can only be used once [14,20].

### 9.4. Phase 4: Representation

The individual clusters and priorities will be entered into the ARIADNE software package and combined to form a group product. The principal component analysis will be used to generate concept maps.

#### 9.4.1. Principal Component Analysis

Principal component analysis (PCA) will be performed to identify hidden patterns in a dataset, reduce the dimensionality of the data by removing the noise and redundancy in the data, and identify correlated variables [24]. PCA will enable us to summarise and to visualise the information in a dataset containing opinions described by multiple inter-correlated quantitative variables [24].

#### 9.4.2. Generation of a Concept Map

Concept maps will be generated, with each statement presented as a singular dot point. The map represents the “distances” between statements: the more often participants put statements in the same category (clustering), the smaller the distance between them on the map. Statements that are rarely or never linked by participants are shown far apart on the map [20]. On the concept map, these “distances” are translated into spatial relationships. We will use “latent preferences” to represent the differences between individual participants and between categories of participants [20].

### 9.5. Phase 5: Interpretation of Concept Maps (via Video Conferencing)

ARIADNE generates up to eight concept maps that need to be qualitatively interpreted and explained [20]. Concept maps produced by ARIADNE will be reviewed by three members of the research team and three participants (via a group video meeting). Participants will be invited to participate in this phase and will be included on a first-come, first-served basis. Researchers and participants will be tasked with coming to a consensus about the concept map that best represents the core competencies of mental health nurses.

The location of the statements on the map will be interpreted to determine which statements or clusters seem to go together logically and which do not [20]. The four cardinal directions of the compass (North, South, East, and West) will guide the interpretation and labelling of the clusters. The statements on the northern part of the map will be interpreted as contrasting with those on the southern aspect. The same applies to the western and eastern parts of the map [20].

The width of the line around each cluster will be used to establish the relative importance which participants attached to each cluster. The thicker the outline, the higher the average priority given to the statements in that cluster [20]. The relative importance of each cluster will be calculated using prioritising data (mean, SD, and 95% CI) [18]. We will generate a table of clusters by rank of importance that will inform an interpretation of the ranking of the relative importance of different clusters of competencies of mental health nurses.

### 9.6. Phase 6: Utilisation of Concept Maps

We will report how we will use the concept maps to inform the curriculum of mental health nurse training internationally.

## 10. The Time Commitment Involved

Typically, participant involvement in the study will take between one and a half and two hours. The brainstorming task takes around 30 min and prioritising and clustering an additional hour. If participants take part in interpreting concept maps, this will take an additional hour.

## 11. Limitations

Our study will be limited by the sampling methods we will use. Sampling and selection biases are inherent inconveniences and snowball sampling [25]. Participants in the population of interest do not have the same probability of being included in the final sample [26]. Sample diversity cannot be achieved through snowball techniques, which is a necessary condition for valid research findings. For example, the participants least keen to cooperate or with a different view are less likely to be referred for an interview [27,28].

There is a possibility that if participants have worked with researchers previously, this may introduce the potential for social desirability bias; this is an acknowledged limitation of this study.

## 12. Conclusions

The views and opinions of the users of mental health services, psychiatric nurses, and psychiatrists on the core competencies of mental health nurses are essential in informing the focus and content of mental health nurse education internationally. Based on our review of the literature, there is a clear gap in knowledge about mental health nurse contemporary core competencies. Our study uses a true mixed-methods approach to develop a sophisticated conceptualisation of mental health nursing competencies from multi-dimensional perspectives. We intend to identify the core competencies of mental health nurses from five separate stakeholder groups and develop a meaningful list of core competencies for mental health nurses.

## Supplementary Materials

The following are available online at https://www.mdpi.com/2039-4403/10/2/18/s1, File S1: The Good Reporting of A Mixed Methods Study (GRAMMS) checklist.

## References

[1] Fukada M. (2018). Nursing Competency: Definition, Structure and Development. Yonago Acta Med..

[2] Bateman J., Henderson C., Kezelman C. (2013). Trauma-Informed Care and Practice: Towards a Cultural Shift in Policy Reform across Mental Health and Human Services in Australia.

[3] Purtle J. (2018). Systematic Review of Evaluations of Trauma-Informed Organizational Interventions That Include Staff Trainings. Trauma Violence Abus..

[4] Howard L.M., Gamble C. (2010). Supporting mental health nurses to address the physical health needs of people with serious mental illness in acute inpatient care settings. J. Psychiatr. Ment. Health Nurs..

[5] Blythe J., White J. (2012). Role of the mental health nurse towards physical health care in serious mental illness: An integrative review of 10 years of UK Literature. Int. J. Ment. Health Nurs..

[6] Slemon A., Jenkins E., Bungay V. (2017). Safety in psychiatric inpatient care: The impact of risk management culture on mental health nursing practice. Nurs. Inq..

[7] Kudless M.W., White J.H. (2007). Competencies and Roles of Community Mental Health Nurses. J. Psychosoc. Nurs. Ment. Health Serv..

[8] Glantz A., Örmon K., Sandström B. (2019). “How do we use the time?”—An observational study measuring the task time distribution of nurses in psychiatric care. BMC Nurs..

[9] Kieft R.A.M.M., De Brouwer B.B.J.M., Francke A.L., Delnoij D.M.J. (2014). How nurses and their work environment affect patient experiences of the quality of care: A qualitative study. BMC Health Serv. Res..

[10] Bee P., Playle J.F., Lovell K., Barnes P., Gray R., Keeley P. (2008). Service user views and expectations of UK-registered mental health nurses: A systematic review of empirical research. Int. J. Nurs. Stud..

[11] Delaney K.R., Johnson M.E. (2014). Metasynthesis of Research on the Role of Psychiatric Inpatient Nurses: What is important to staff?. J. Am. Psychiatr. Nurses Assoc..

[12] Rosas S.R., Kane M. (2012). Quality and rigor of the concept mapping methodology: A pooled study analysis. Eval. Program. Plan..

[13] Kane M., Trochim W. (2007). Concept Mapping for Planning and Evaluation.

[14] Trochim W.M. (1989). An introduction to concept mapping for planning and evaluation. Eval. Program. Plan..

[15] O’Cathain A., Murphy E., Nicholl J. (2008). The Quality of Mixed Methods Studies in Health Services Research. J. Health Serv. Res. Policy.

[16] A Johnsen J., E Biegel D., Shafran R. (2000). Concept mapping in mental health: Uses and adaptations. Eval. Program. Plan..

[17] Burke J.G., O’Campo P., Peak G.L., Gielen A.C., McDonnell K.A., Trochim W.M.K. (2005). An Introduction to Concept Mapping as a Participatory Public Health Research Method. Qual. Health Res..

[18] Kikkert M.J., Schene A.H., Koeter M.W.J., Robson D., Born A., Helm H., Nosè M., Goss C., Thornicroft G., Gray R. (2006). Medication Adherence in Schizophrenia: Exploring Patients’, Carers’ and Professionals’ Views. Schizophr. Bull..

[19] Brown E., Topping A., Cheston R. (2019). What are the barriers to accessing psychological therapy in Qatar: A concept mapping study. Couns. Psychother. Res..

[20] Ariadne (2015). Manual Ariadne 3.0. http://www.minds21.org/images_public/manual%20%20ARIADNE%203.0%20%20april%202015.pdf.

[21] Rid A., Wendler D. (2010). Risk-benefit assessment in medical research—Critical review and open questions. Law Probab. Risk.

[22] Driggin E., Madhavan M.V., Bikdeli B., Chuich T., Laracy J., Biondi-Zoccai G., Brown T.S., Der Nigoghossian C., Zidar D.A., Haythe J. (2020). Cardiovascular Considerations for Patients, Health Care Workers, and Health Systems during the COVID-19 Pandemic. J. Am. Coll. Cardiol..

[23] Cirrincione L., Plescia F., Ledda C., Rapisarda V., Martorana D., Moldovan R.E., Theodoridou K., Cannizzaro E. (2020). COVID-19 Pandemic: Prevention and Protection Measures to Be Adopted at the Workplace. Sustainability.

[24] Kassambara A. (2017). Practical Guide to Principal Component Methods in R: PCA, M (CA), FAMD, MFA, HCPC, Factoextra (Vol. 2). http://www.sthda.com/english/articles/31-principal-component-methods-in-r-practical-guide/112-pca-principal-component-analysis-essentials/.

[25] Palinkas L.A., Horwitz S.M., Green C.A., Wisdom J.P., Duan N., Hoagwood K. (2015). Purposeful Sampling for Qualitative Data Collection and Analysis in Mixed Method Implementation Research. Adm. Policy Ment. Health Ment. Health Serv. Res..

[26] Kirchherr J., Charles K. (2018). Enhancing the sample diversity of snowball samples: Recommendations from a research project on anti-dam movements in Southeast Asia. PLoS ONE.

[27] Penrod J., Preston D.B., Cain R.E., Starks M.T. (2003). A Discussion of Chain Referral as a Method of Sampling Hard-to-Reach Populations. J. Transcult. Nurs..

[28] Heckathorn D.D., Semaan S., Broadhead R.S., Hughes J.J. (2002). Extensions of Respondent-Driven Sampling: A New Approach to the Study of Injection Drug Users Aged 18–25. Aids Behav..

