# Supplementary material for: What Are the Core Competencies of a Mental Health Nurse? Protocol for a Concept Mapping Study
Source: Nurs Rep. 2020 Dec 7;10(2):146–53. doi: 10.3390/nursrep10020018 (PMC8608074; doi:10.3390/nursrep10020018)
Supplement: Supplementary file 1 [file nursrep-10-00018-s001.pdf]

Supplementary File S1: The Good Reporting of A Mixed Methods Study (GRAMMS) checklist
This checklist was adapted from O'Cathain, Murphy, Nicholl (15).

**Guidelines**	**Section: page**
1. Describe the justification for using a mixed-methods approach to the research question	Material and methods – under why concept mapping Page 3
2. Describe the design in terms of the purpose, priority and sequence of methods	Material and methods – under concept mapping and data collection procedure Page 2 to 5
3. Describe each method in terms of sampling, data collection and analysis	Material and methods – under concept mapping and data collection procedure Page 2 to 7
4. Describe where integration will occur, how it will occur and who will participate in it	Material and methods – concept mapping Page 2 & 7
5. Describe any limitation of one method associated with the presence of the other method	Material and methods – concept mapping Page 3
6. Describe any insights gained from mixing or integrating methods	Conclusion- Page 8
